# Is there clinical evidence to support alveolar ridge preservation over extraction alone? A review of recent literature and case reports of late graft failure

**DOI:** 10.1038/s41415-022-4967-2

**Published:** 2022-09-23

**Authors:** Robert J. Adams

**Affiliations:** Senior Clinical Lecturer, Dental Implantology, Cardiff University, Cardiff, UK

## Abstract

Since its introduction in 1998, alveolar ridge preservation has become a popular technique, currently accounting for approximately 29% of all procedures involving bone substitute materials. The global cost of bone substitute materials for alveolar ridge preservation is estimated at $190 million annually and is expected to rise by approximately 11.4% per year.

Numerous randomised controlled trials have compared alveolar ridge preservation to extraction alone. A recent Cochrane review reported that, in terms of socket dimensional change, the mean difference between alveolar ridge preservation and extraction alone is 1.18 mm horizontally and 1.35 mm vertically. The clinical impact of this is uncertain, for there is no significant difference in the need for graft procedures at implant placement between ridge preservation and extraction alone. There are no randomised controlled trials comparing aesthetic or functional outcomes.

A systematic review of the histological outcomes of ridge preservation demonstrates that, compared to extraction alone, many bone substitute materials can significantly delay the bone healing process. No bone substitute material achieves statistically more new bone formation than extraction alone and many commonly used materials achieve significantly less bone formation. Grafted sites can demonstrate high levels of residual graft and granulation tissue.

In the absence of good-quality clinical evidence to support alveolar ridge preservation, the technique must be questioned as the treatment of choice at extraction sites. This paper assesses recent systematic reviews and presents two case reports of late graft failure.

## Key points

- Current high-level evidence shows that alveolar ridge preservation has minimal effect at preventing post-extraction remodelling.
- Histological evidence shows that many bone substitute materials can significantly impair new bone formation.
- There is no evidence that alveolar ridge preservation improves clinical outcomes of implant treatment.

## Introduction

The use of bone substitute materials (BSM) in oral surgery and implantology has been reported since the early 1970s, when hydroxyapatite was first used in the replacement of missing bone tissue.^1^ Initially, the use of BSM was restricted to tissue replacement before or at the time of implant placement. Subsequently, there has been an increase in applications and current usage includes gap fill at immediate implant placement,^2^ guided bone regeneration/contour augmentation,^3^ alveolar ridge preservation,^4^ partial extraction technique,^5^ lateral ridge augmentation,^6^ vertical ridge augmentation,^7^ maxillary sinus grafting,^8^ peri-implantitis defects,^9^ periapical surgery,^10^ cyst cavity defects,^11^ periodontal regenerative therapy,^12^ periodontally accelerated osteogenic orthodontics^13^ and root coverage surgery.^14^

The global market for BSM was valued at $663.2 million in 2020 and is expected to have a compound annual growth rate of 11.4% from 2021-2028.^15^ Xenografts are currently the most frequently used BSM, accounting for 47% of grafts in 2020. Alveolar ridge preservation (ARP) has become the most frequent BSM procedure, at 29% of all grafts.^15^ The aim of this paper is to review recent systematic review and meta-analysis studies of alveolar ridge preservation and present case reports of late graft failure following these procedures.

## Bone substitute materials

Many BSM are available as alternatives to autogenous bone in implantology/oral surgery. The commonly used BSM fall into four categories: allograft; xenograft; alloplast; and biomodulatory therapies. Allograft is derived from a donor from the same species as the recipient; xenograft is derived from a different species (for example, bovine/porcine); alloplast is synthetically manufactured material; and biomodulatory therapies utilise growth factors typically derived from autologous blood. Examples are plasma-rich in growth factors, platelet-rich fibrin (PRF), bone morphogenic protein and platelet-rich plasma.

In terms of their clinical properties, BSM can be classified as resorbable (with typical resorption times of 1-6 months) or non-resorbable.

## Alveolar ridge preservation

The earliest reports of ARP used hydroxyapatite at extraction sites to improve the retention of full dentures.^16^ The use of ridge preservation before dental implant placement was first reported by Artzi and Nemcovsky in 1998.^4^ It has been defined as 'preserving the ridge volume within the envelope existing at the time of extraction'.^17^ The technique involves minimally traumatic tooth extraction followed by immediate grafting of the extraction sockets, utilising particulate bone grafts or substitutes.^18^ The graft is typically left for up to six months before implant placement. The stated rationale for ARP is to preserve or minimise post-extraction bone loss to allow optimal implant placement so that the final implant aesthetic and functional outcomes are not compromised.^4^ Several authors have presented clinical recommendations/guidelines for ARP based on extraction socket morphology.^19^^,^^20^^,^^21^

Elian *et al.*^19^ classified extraction sockets with the following morphology:

- Type 1 socket - the facial soft tissue and buccal plate are at normal levels in relation to the cementoenamel junction of pre-extracted teeth and remain intact post extraction
- Type 2 socket - facial soft tissue is present but the buccal plate is partially missing following extraction of the tooth
- Type 3 socket - the facial soft tissue and the buccal plate of bone are markedly reduced after tooth extraction.

Elian *et al.* recommended ARP for Type 2 sockets where the facial soft tissue level is present but the buccal plate is partially missing.^19^ Jung *et al.* recommended ARP in cases with severe loss of the buccal plate (>50% bone loss) when early, Type 2, implant placement (6-8 weeks) is contraindicated.^20^ Juodzbalys *et al.* recommended ARP when immediate implant placement is not possible in the following situations: sufficient aesthetic result can't be achieved; impossible to gain primary stability; risk of significant alveolar bone resorption; risk of apical peri-implantitis; or risk of maxillary sinus or nasal floor perforation reducing the need for elevation of the maxillary sinus or nasal floor.^21^

## Socket seal surgery

Socket seal surgery (SSS) was first described in 1994 by Landsberg and Bichacho.^22^ The aim of SSS is soft tissue preservation at extraction sites using connective tissue grafts or biomaterials.^23^ It has been defined as 'a procedure that, through soft tissue grafts or biomaterials, can seal the socket, complementing the guided bone regeneration or acting alone to preserve the soft tissues, thereby preventing its collapse'.^23^ SSS techniques include the use of free gingival grafts, collagen matrix, collagen sponge and acellular dermal matrices.^23^ SSS can be undertaken to complement guided bone regeneration or alone.^23^

## Graft outcome measures

Several methods have been used in the assessment of graft success. These include dimensional change, radiographic appearance, implant survival rates, periodontal parameters (bleeding scores, plaque scores, probing pocket depths) and patient-reported outcome measures.

## Socket dimensional change

Several systematic review and meta-analysis studies have reported on the dimensional change of extraction sites with and without ARP.^18^^,^^24^^,^^25^^,^^26^^,^^27^ All studies reporting dimensional change demonstrate a statistically significant reduction in socket dimensional change following ARP compared to extraction alone, although there is variation in the results. Avila-Ortiz *et al.* reported a mean difference of 1.99 mm in a horizontal direction (based on 11 randomised controlled trials [RCTs]) and 1.72 mm vertically (based on 12 RCTs).^26^ This study does, however, include cases of SSS as well as ARP.^26^ Bassir *et al.* reported on 14 studies (11 RCTs/three controlled clinical trials [CCTs]).^25^ They found a horizontal mean difference of 1.86 mm and vertical difference of 1.55 mm.^25^ MacBeth *et al.* in a systematic review of eight RCTs and one CCT reported a mean horizontal difference of 1.198 mm and vertical mean difference of 0.739 mm.^18^ The most recent Cochrane review was published in 2021.^27^ Inclusion criteria were RCTs of at least six-month follow-up. In total, 16 studies met the inclusion criteria (524 extraction sites in 426 participants). Four of these studies were reported to be high risk of bias and the remainder had unclear risk of bias. Seven of the RCTs compared dimensional change of ARP to extraction alone (201 extraction sites in 184 participants). Although statistically significant, the mean difference between ARP grafted sites and extraction alone was 1.18 mm (0.54-1.82 mm) in a horizontal direction and 1.35 mm (0.70-2.00 mm) in a vertical direction.^27^

Some authors have tried to relate the results of dimensional change to the pre-extraction socket morphology.^25^^,^^26^ Avila-Ortiz *et al.* found that the results of ARP were significantly more favourable in patients with thick buccal plate (>1 mm) compared to those with thin buccal plate (<1 mm).^26^ In patients with thick buccal plate, the horizontal mean difference between ARP and extraction alone was 3.2 mm but in patients with thin buccal plate, the mean difference was only 1.29 mm.^26^ Bassir *et al.* reported a horizontal mean difference of 2.88 mm when extraction sockets were intact but only 1.71 mm when sockets were damaged.^25^

Other authors have tried to look at the influence of different graft materials on dimensional change.^24^^,^^28^^,^^29^ A recent systematic review identified 31 RCTs of 25 different BSM.^29^ Of these, only eight RCTs reported a significant mean difference in dimensional change compared to extraction alone. Of these eight RCTs, five of the BSMs used were xenograft (Aptos, Bio-Oss, Bio-Oss/Collagen, Gen-Os and mp3), one was allograft (freeze-dried bone allograft [FDBA]), one was synthetic hydroxyapatite (Bond Apatite) and one study used leukocyte-PRF.^29^ Other than leukocyte-PRF, no resorbable BSM was found to have a significant mean difference in socket dimensional change compared to extraction alone.^29^

## Histological outcome

Several studies have reported the mean new bone formation (NBF) after 12 weeks at extraction sites. There is variation in reported results with NBF at 12 weeks post-extraction, ranging between 41.5-52.1%.^30^^,^^31^ A 2020 systematic review reported on 38 RCTs where core biopsies had been taken 3-6 months following ARP.^32^ The study reported meta-analysis from 33 studies with new bone formation (NBF) observed by histological samples taken at implant placement (1,268 extraction sites in 985 patients).^32^

The authors reported that none of the biomaterials had significantly more NBF compared to extraction alone. Eight of the RCTs demonstrated sites with significantly less new bone formation than extraction alone. All of these eight RCTs utilised non-resorbable BSMs (five xenograft/two allograft/one alloplast). Extractions sites filled with Bio-Oss had on average 22.5% (14.7-30.0%) less NBF than extraction alone. Sites filled with FDBA had on average 22.0% (10.3-34.1%) less NBF. In a further 15 studies, sites filled with BSM had less NBF than extraction alone but the results were not statistically significant. Ten studies reported the same or more NBF than extraction alone, although none of these results were statistically significant.^32^

Previous histological studies of ARP have demonstrated highly variable outcomes of treatment.^31^^,^^33^ Carmagnola *et al.* (2003) studied histological core biopsies taken at seven months after ARP with Bio-Oss.^31^ The results showed mean lamellar bone formation of 26 ± 23.7%, woven bone 8.4 ± 8.0%, connective tissue 18.1 ± 17.0% and residual graft 21 ± 20%. Contact between graft and surrounding bone was also found to be highly variable. The mean Bio-Oss to bone contact was 40.3 ± 37.2%. Sites demonstrating significantly less new bone formation, can present a risk of chronic inflammation. Rodriguez and Nowzari (2019) published a case series of long-term complications of xenografts up to 13 years following treatment.^34^ These complications included fibrous encapsulation and chronic infection of the BSM.^34^

**Fig. 1:**
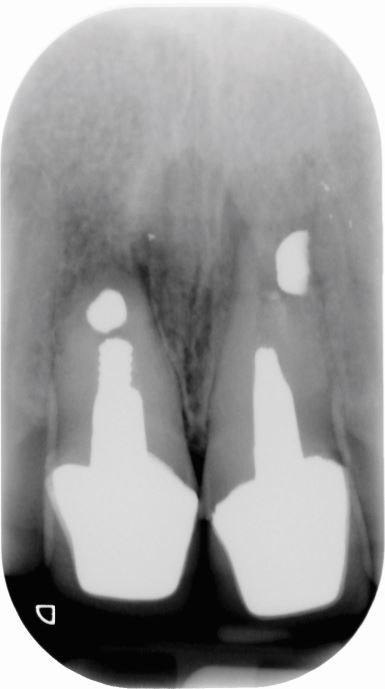
Pre-extraction of the 11 and 21

**Fig. 2:**
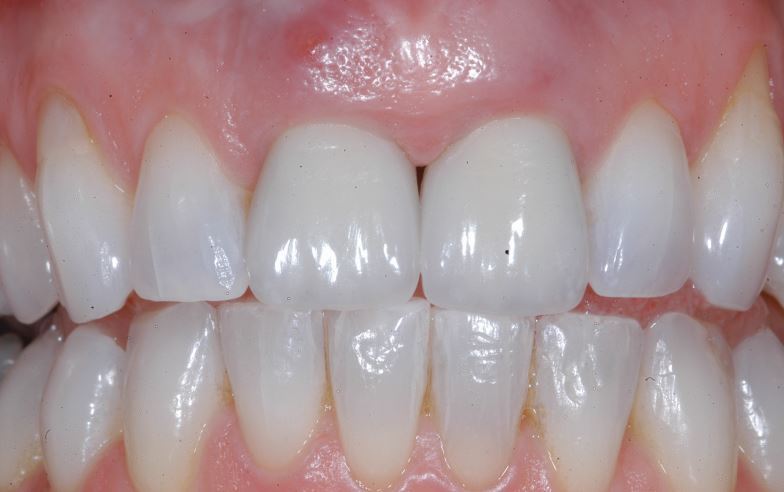
Implant restoration of the 11 with buccal sinus tract

**Fig. 3:**
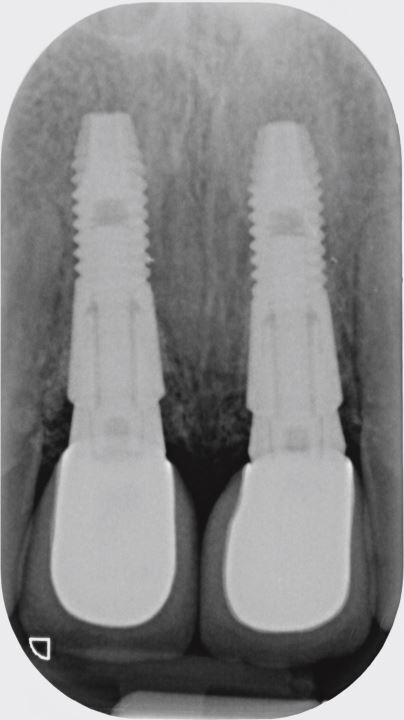
Radiograph showing apparent good radiographic bone levels

**Fig. 4:**
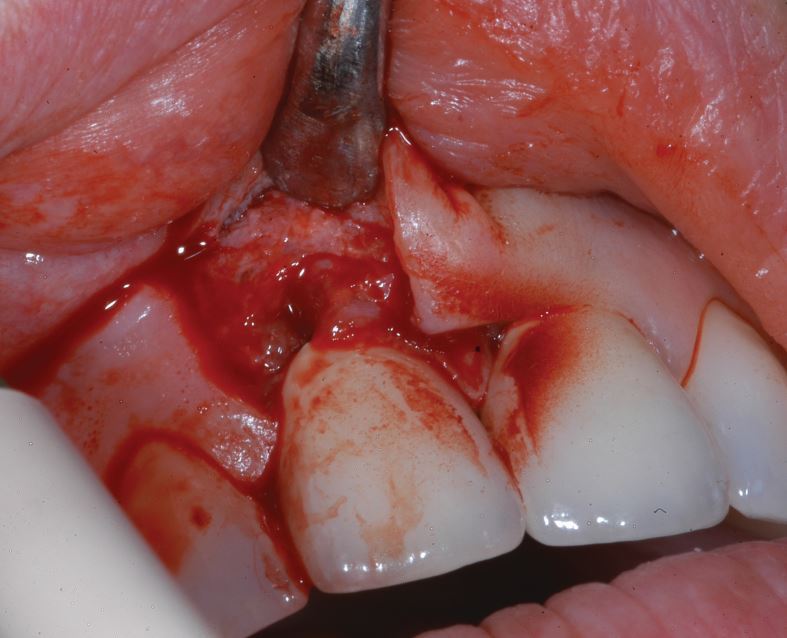
Surgical exposure showing granulation tissue/BSM

**Fig. 5:**
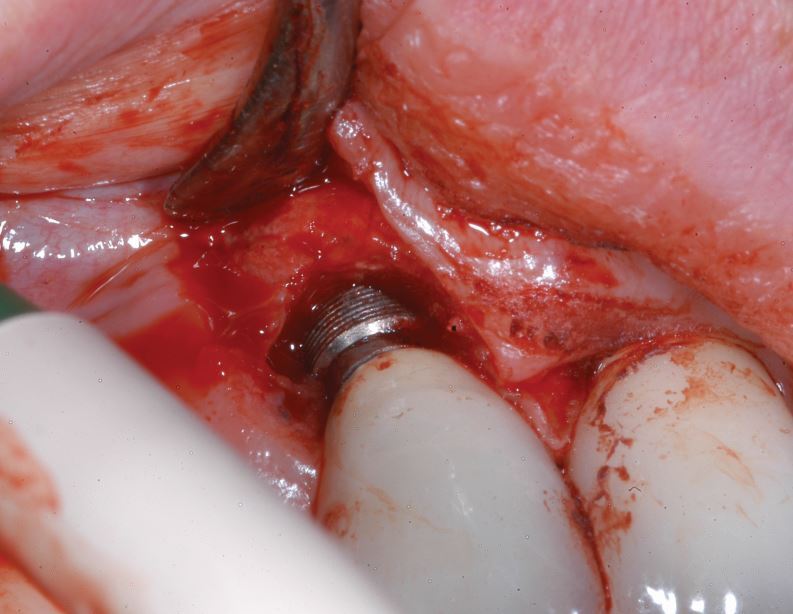
Surgical debridement of graft material/granulation tissue

**Fig. 6:**
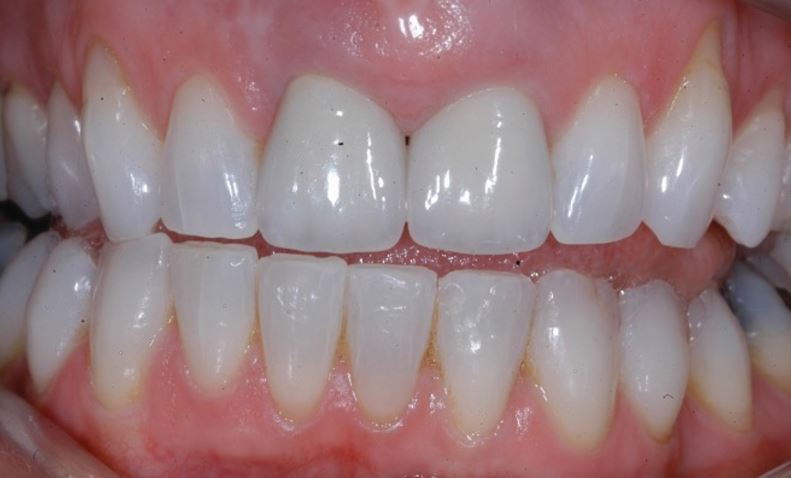
12-month follow-up showing acceptable soft tissue level of the 11

Figures 1, 2, 3, 4, 5 and 6 illustrate a clinical case of late graft failure, undertaken in 2012. The patient presented with failing apicected post crowns of the 11 and 21 (Fig. 1). The patient was about to commence pre-operative orthodontics and was advised to have ARP with a xenograft bone substitute as implant placement was going to be delayed by the orthodontic treatment. At the time of implant placement, it was recorded that the bone quality was 'soft'. At a check-up appointment, 12 months after restoration, a draining sinus tract was identified at the 11 (Fig. 2). Radiographs suggested some non-integrated graft material (Fig. 3). Surgical exposure identified non-integrated xenograft embedded in granulation tissue (Fig. 4). This was debrided and the area allowed to heal naturally (Fig. 5). The soft tissue levels have remained stable following that treatment (Fig. 6).

**Fig. 7:**
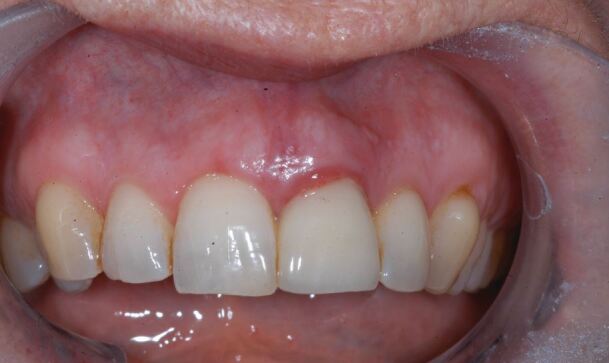
Clinical presentation of 'peri-implantitis' of the 21

**Fig. 8:**
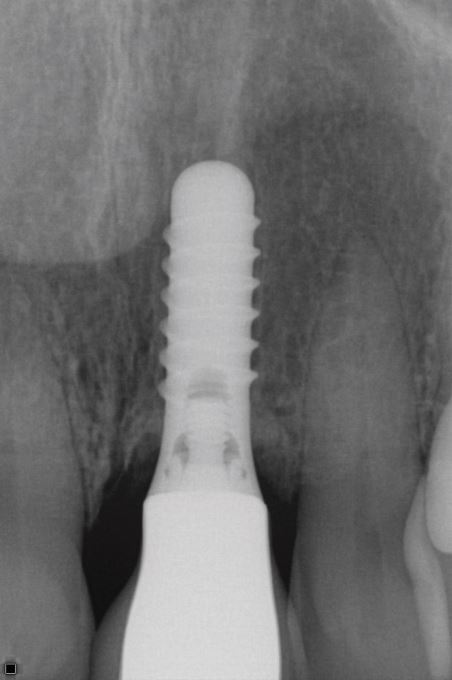
Intraoral periapical radiograph of 21 showing apparent good radiographic bone level

**Fig. 9:**
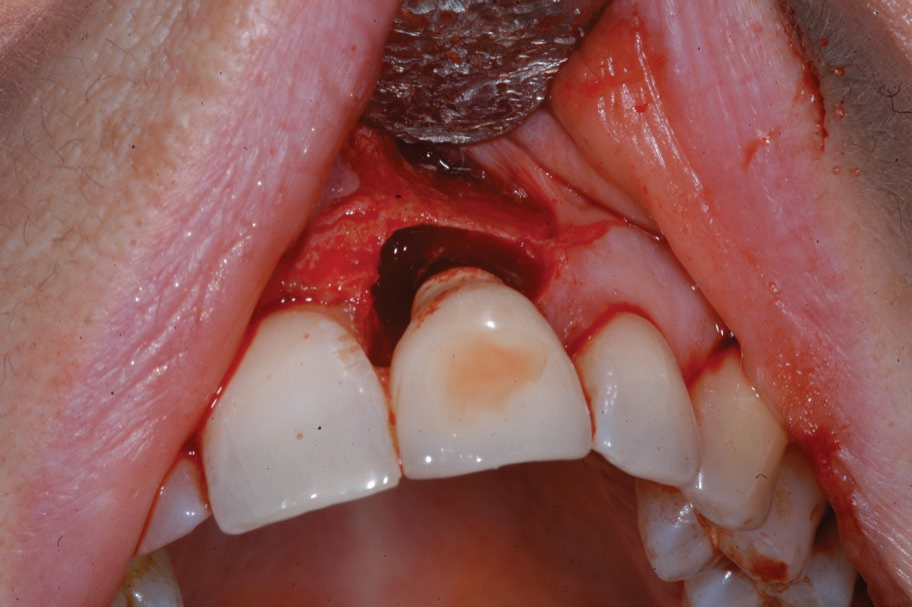
Surgical debridement of non-integrated graft material showing bone defect of the 21

**Fig. 10:**
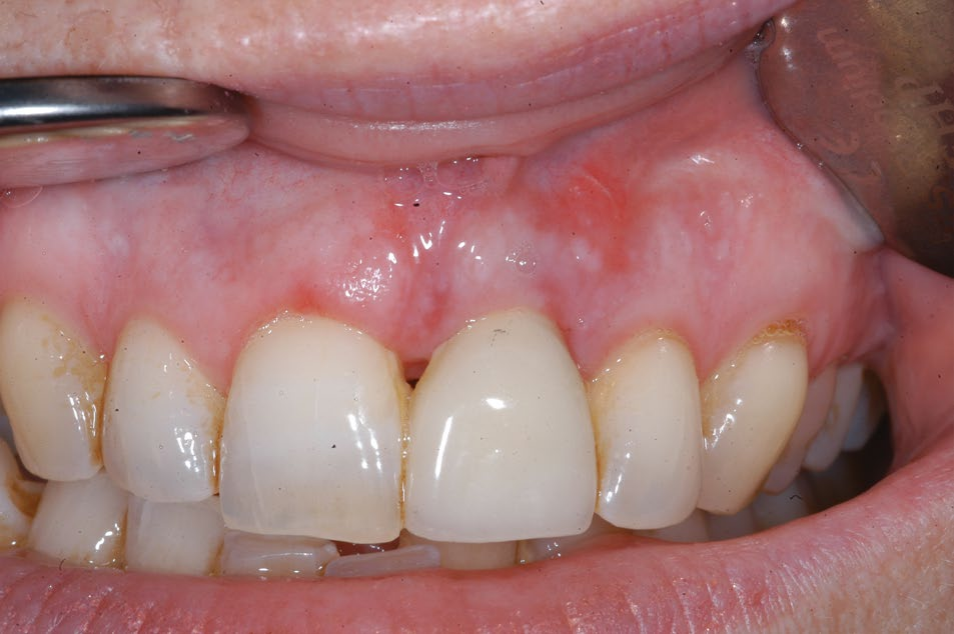
Two weeks post surgical debridement of the 21 showing marked recession

**Fig. 11:**
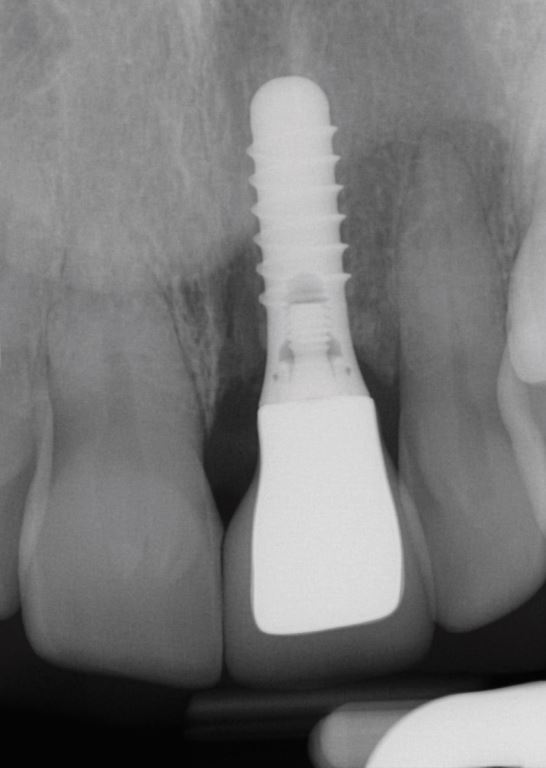
Intraoral periapical radiograph two weeks post debridement showing radiographic bone defect

**Fig. 12:**
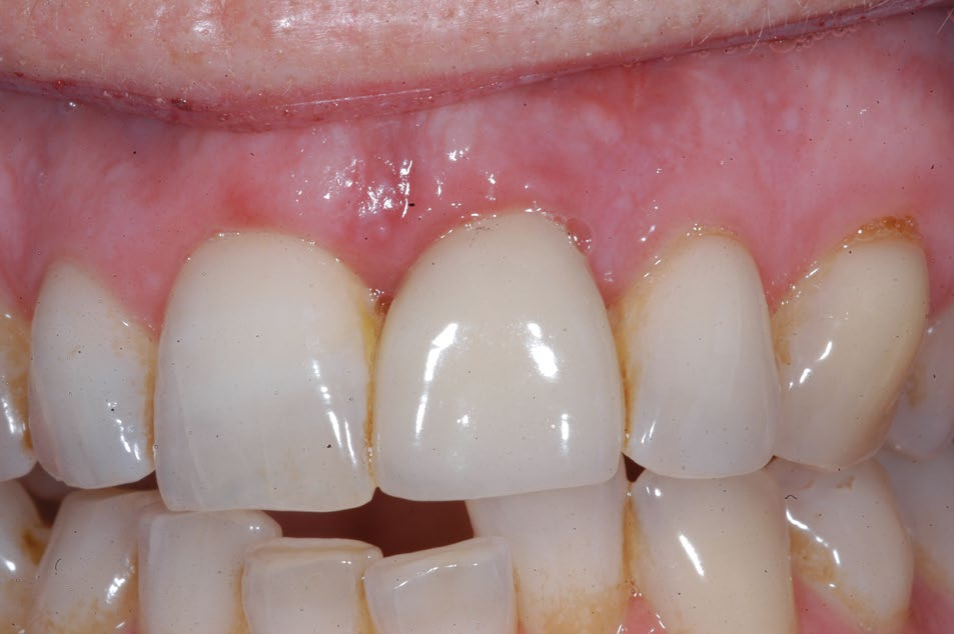
Six months post surgical debridement showing soft tissue rebound

**Fig. 13:**
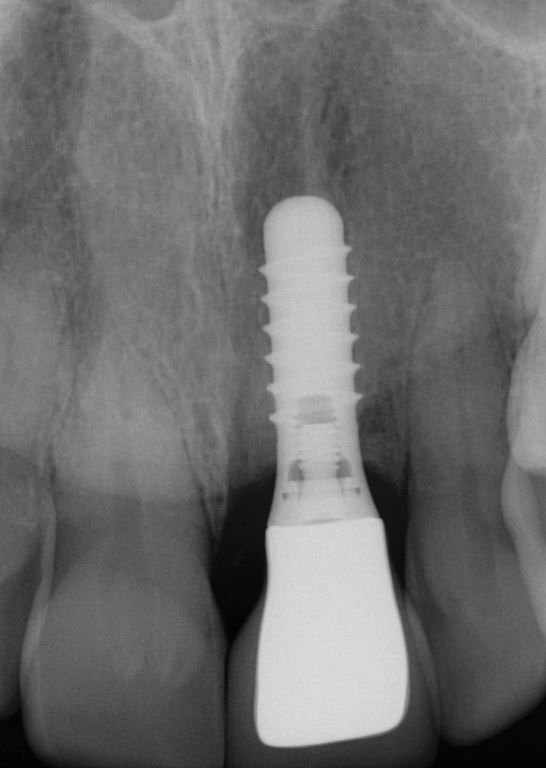
Intraoral periapical radiograph six months post debridement showing apparent radiographic bone infill

The second case (Figures 7, 8, 9, 10, 11, 12 and 13) was referred to the author, in 2017, for the treatment of peri-implantitis. The patient presented with an implant at the 21 that had been undertaken following ARP at least ten years previously (Fig. 7). While clinical examination demonstrated swollen and erythematous gingivae with bleeding on probing consistent with peri-implantitis, the intraoral periapical showed no apparent bone loss (Fig. 8). Exploration found non-integrated graft material which was surgically debrided (Fig. 9). Early post-op review found marked soft tissue recession (Fig. 10, Fig. 11). At six-months follow-up, there had been some rebound of the soft tissues and the radiographic appearance of bone infill (Fig. 12, Fig. 13). The implant has remained asymptomatic since the cleaning procedure in 2017. The surgically debrided material was sent for histology. The histological report was 'inflamed mucosa and granulation tissue with mixed inflammation. Some calcified material is present but its precise nature is unclear'.

## Clinical outcomes

Despite the statistically significant dimensional change, systematic reviews have failed to show any significant difference in clinical outcomes between ARP and extraction alone.

The recent Cochrane review reported on four randomised controlled trials (154 participants/156 extraction sites) that studied the need for additional augmentation at implant placement.^27^ The authors found that there was no significant difference between the two groups. Sites that have received ARP are no less likely to need further augmentation than sites that have received no augmentation at the time of extraction (risk ratio 0.68; p = 0.39).^27^

No RCTs have compared the aesthetic outcome, implant failure, peri-implant marginal bone level changes, changes in probing depth or prosthodontic outcomes.^27^

## Conclusions

The increased popularity of alveolar ridge preservation, over extraction alone, does not appear to be based on clinical evidence. Current evidence, based on RCTs, shows that the mean difference in dimensional change is 1.18 mm horizontally and 1.35 mm vertically. The clinical impact of this is unproven. Other than leukocyte-PRF, no resorbable BSM has been shown to have any difference in socket dimensional change compared to extraction alone. Clinical studies show that ARP does not prevent the need for further augmentation at implant placement and there are no studies comparing aesthetic/functional outcomes. Histological studies show that many BSM (mostly xenograft/allograft) can be associated with significantly less new bone formation than extraction alone. There is a risk following ARP that extraction sites can contain high levels of residual graft and granulation tissue. There does not appear to be a clear clinical benefit of ARP and large-scale, clinically relevant RCTs are needed to demonstrate clinical advantages of this procedure.
